# Unveiling Donor-Derived BKPyV DNAemia Through Analysis of Contralateral Kidney Transplant Recipients

**DOI:** 10.3390/biomedicines14040820

**Published:** 2026-04-03

**Authors:** Wouter T. Moest, A. Lianne Messchendorp, Helma Dolmans, Cynthia Konijn-Janssen, Stan van den Eijnden, Milou van Bruchem, Ineke Tieken, Maarten H. L. Christiaans, Arjan D. van Zuilen, Marcia M. L. Kho, Irma Stijnman, Frederike J. Bemelman, Jan-Stephan S. Sanders, Mariet C. W. Feltkamp, Aiko P. J. de Vries, Joris I. Rotmans

**Affiliations:** 1 Department of Internal Medicine, Leiden University Medical Center (LUMC), 2333 ZA Leiden, The Netherlands; 2 Department of Medical Microbiology and Infection Prevention, University Medical Center Groningen (UMCG), 9713 GZ Groningen, The Netherlands; 3 Department of Nephrology, University Medical Center Utrecht (UMCU), 3584 CX Utrecht, The Netherlands; 4 Nederlandse Transplantatie Stichting (NTS), 2332 KG Leiden, The Netherlands; 5 EuroTransplant, 2332 KG Leiden, The Netherlands; 6 Department of Internal Medicine, Division of Nephrology, Maastricht University Medical Center (MUMC), 6229 HX Maastricht, The Netherlands; 7 Department of Internal Medicine, Section of Nephrology & Transplantation, Erasmus Medical Center (EMC), 3015 GD Rotterdam, The Netherlands; 8 Department of Internal Medicine, Amsterdam University Medical Center (AUMC), 1105 AZ Amsterdam, The Netherlands; 9 Department of Internal Medicine, Division of Nephrology, University Medical Center Groningen (UMCG), 9713 GZ Groningen, The Netherlands; 10 Department of Medical Microbiology & Infection Prevention, Leiden University Center of Infectious Diseases, Leiden University Medical Center (LUMC), 2333 ZA Leiden, The Netherlands

**Keywords:** BK polyomavirus, kidney transplantation, donor-derived infection, viral transmission

## Abstract

**Background:** BK polyomavirus (BKPyV) infection is of notable concern in kidney transplant recipients, as it can cause BKPyV-associated nephropathy (BKPyVAN). Currently, there is no effective treatment for BKPyV infection, underscoring the need for preventive strategies. There is emerging evidence that donor-derived BKPyV plays a role in the development of BKPyV DNAemia. To further explore this hypothesis, we conducted a retrospective, multi-center cohort study to evaluate the risk of developing BKPyV DNAemia in kidney recipient pairs sharing the same donor. **Methods:** At the Leiden University Medical Center (LUMC), deceased donor kidney transplant recipients (2011–2021) were identified and classified according to the occurrence of BKPyV DNAemia within the first year post-transplantation. For each recipient, the contralateral kidney recipient from the same donor was identified through national transplant registries. Cox regression was used to assess whether BKPyV DNAemia in the LUMC recipient was associated with an increased risk of BKPyV DNAemia in the contralateral recipient. **Results:** Among 117 recipient pairs, BKPyV DNAemia was more frequent when the contralateral recipient was affected (28.8% [15/52]), compared with pairs in which the contralateral recipient remained unaffected (10.8% [7/65], *p* = 0.013). Multivariable Cox regression analysis confirmed this increased risk (HR 4.9, 95% CI: 1.8–13.6; *p* = 0.002). **Conclusions:** This study shows a significantly increased risk of BKPyV DNAemia in recipients of deceased donor kidneys when the contralateral kidney recipient develops BKPyV DNAemia. These findings highlight the influence of donor-derived factors in BKPyV transmission in kidney transplantation.

## 1. Introduction

BK polyomavirus (BKPyV) infection is of notable concern in kidney transplant recipients, as it can lead to BKPyV DNAemia and BKPyV-associated nephropathy (BKPyVAN). BKPyVAN can cause significant damage to the transplanted kidney, potentially resulting in graft loss [1,2,3]. Currently, there is no effective treatment against BKPyV, underscoring the need for preventive strategies. Identifying and addressing risk factors for BKPyV DNAemia is essential, with donor-derived BKPyV emerging as a key factor.

There are several indications that donor-derived BKPyV plays an important role in the development of BKPyV infections. One relevant observation is that the incidence of BKPyV DNAemia is notably higher in kidney transplant recipients (10–30%) compared to those who receive non-renal solid organ transplants (0–3%), despite the use of comparable induction and maintenance immunosuppressive regimens [2,3,4,5,6,7,8]. This could suggest that in the case of kidney transplants, BKPyV residing in the kidney may be co-transplanted with the organ. The recipient might then be exposed to a higher viral load or a more virulent strain of BKPyV, leading to BKPyV-related infections. This is supported by the observation of a strong association between high BKPyV serotiters in kidney donors and the subsequent risk of BKPyV DNAemia in transplant recipients [9]. In line with this hypothesis, previous studies revealed that the *VP1* gene sequences in the urine of 20 donor-recipient pairs were completely identical, underscoring the role that donor-derived BKPyV appears to play in kidney transplant recipients [10].

These studies on donor-derived BKPyV have primarily focused on recipients of living donors, likely due to the absence of pre-transplant donor samples in deceased donors. To address the potential role of donor-derived BKPyV in recipients of deceased donors, we conducted a retrospective, multi-center cohort study, evaluating the risk of developing BKPyV DNAemia in kidney recipient pairs from the same deceased donor. We hypothesize that the likelihood of developing BKPyV DNAemia increases if the recipient of the contralateral kidney also develops BKPyV DNAemia.

## 2. Method

### 2.1. Study Population and Cohort Design

This retrospective study was based on kidney transplant recipient pairs sharing the same deceased donor. At the Leiden University Medical Center (LUMC), we selected all patients who received a Donation after Brain Death (DBD) or Donation after Circulatory Death (DCD) kidney transplant between January 2011 and December 2021 and who developed BKPyV DNAemia within the first year post-transplantation. Patients who developed BKPyV DNAemia following rejection therapy were excluded to minimize confounding by increased immunosuppression. The control group consisted of kidney transplant recipients from a deceased donor who did not develop BKPyV DNAemia within the first year post-transplant. These controls were selected from December 2021 to February 2018 in reverse chronological order until a similar number of controls were identified.

Patients with graft failure within 3 months after transplantation or those who underwent combined organ transplants were excluded from both groups.

For each included LUMC recipient, the recipient of the contralateral kidney from the same donor was identified through Eurotransplant (ET) and Nederlandse Transplantatie Stichting (NTS) registries. If both kidneys from a deceased donor were transplanted and the contralateral kidney was allocated to a Dutch transplantation center, the respective center was contacted for data collection, which included University Medical Center Groningen (UMCG), University Medical Center Utrecht (UMCU), Maastricht University Medical Center (MUMC+), Erasmus Medical Center Rotterdam (EMC), and Amsterdam University Medical Center (AUMC). The collected data included patient characteristics; details on the incidence and course of BKPyV DNAemia; and potential risk factors for BKPyV DNAemia, including induction therapy, maintenance immunosuppression, rejection treatment, use of a ureteral stent, delayed graft function (DGF), and cold ischemia time.

The primary objective was to assess whether the occurrence of BKPyV DNAemia in the LUMC recipient was associated with the risk of BKPyV DNAemia in the recipient of the contralateral kidney from the same donor.

For the purpose of this study, the endpoint of BKPyV DNAemia was reached if there was at least one positive measurement.

### 2.2. BKPyV DNAemia Screening Protocols Across Transplantation Centers

In most transplant centers, BKPyV DNAemia screening is routinely performed during the first year after kidney transplantation. When a screening protocol is in place, BKPyV DNAemia is assessed using quantitative real-time polymerase chain reaction (qPCR) on EDTA–plasma samples. At the LUMC, BKPyV DNAemia screening was performed at 1.5, 3, 6, and 12 months post-transplantation. At the EMC, screening occurred at 3 and 12 months, while at the UMCU, it was conducted at 6 weeks, 3 months, and 12 months. The UMCG and AUMC followed a screening protocol at 3, 6, and 12 months after transplantation. In contrast, the MUMC+ did not have a standardized BKPyV DNAemia screening program, but serum samples collected at 3 and 12 months after transplantation were stored and retrospectively analyzed at LUMC using qPCR for BKPyV DNA, as previously described [9].

### 2.3. Immunosuppressive Regimens Across Transplantation Centers

Immunosuppressive protocols also varied between centers. The LUMC, EMC, UMCU, AUMC, and UMCG followed a standardized regimen, which included basiliximab induction therapy, followed by maintenance immunosuppression with prednisone, tacrolimus, and mycophenolate mofetil. In contrast, the MUMC+ applied a minimized immunosuppressive strategy, initiating therapy with tacrolimus, mycophenolate mofetil, and prednisone (10 days in low immunological risk transplants) without induction therapy. Based on clinical evaluation and a protocol kidney biopsy at three months, immunosuppression was reassessed, with the potential for discontinuing prednisone and mycophenolate mofetil.

### 2.4. Statistical Analysis

IBM SPSS Statistics version 25 was used for statistical analysis. To compare patient characteristics between groups (transplanted at the LUMC without BKPyV DNAemia vs. transplanted at the LUMC with BKPyV DNAemia; contralateral kidney recipients with BKPyV DNAemia vs. without BKPyV DNAemia), the chi-square test and independent-sample *t*-test were employed. When cell counts were <5, Fisher’s exact test was applied. Univariable and multivariable Cox regression analyses were performed to evaluate whether BKPyV DNAemia in the LUMC recipient was associated with the risk of BKPyV DNAemia in the contralateral kidney recipient. Candidate risk factors included recipient age and gender, donor age and gender, panel-reactive antibody (PRA), HLA mismatches, induction therapy, initial maintenance immunosuppression regimen, rejection within the first year of transplantation, transplant type (DBD/DCD), cold ischemic time, warm ischemic time, the use of a ureteral stent or splint, and the length of stay. Additionally, interactions between PRA and induction therapy, PRA and maintenance immunosuppression, rejection treatment and PRA, and maintenance immunosuppression and induction therapy were assessed. Variables were included in the final multivariate analysis based on clinical relevance and statistical significance. For all the performed tests, a *p*-value < 0.05 in a two-sided test was considered statistically significant.

### 2.5. Sensitivity Analysis

To assess the robustness of the primary findings and specifically address potential confounding due to center-specific immunosuppressive strategies and differences related to time periods between groups, sensitivity analyses were performed. First, analyses were repeated after exclusion of recipients transplanted at the MUMC+, given its less intensive immunosuppressive regimen compared with other centers. Second, to account for time-period-related confounding, analyses were restricted to all deceased donor kidney transplantations at the LUMC between 2018 and 2021. For both sensitivity analyses, univariate and multivariable Cox regression models were applied using the same covariates as in the primary analysis.

The study protocol was submitted to the Medical Ethics Committee of the LUMC, EMC, UMCG, UMCU, AUMC, and MUMC+, who decided formal approval was not needed due to the retrospective study design.

## 3. Results

**Figure 1:**
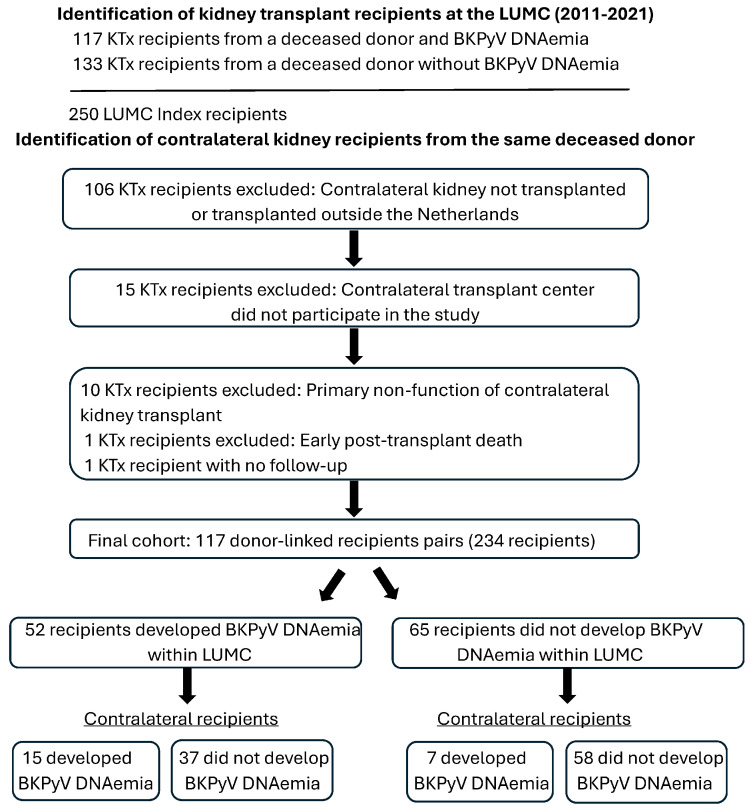
Flowchart of patient inclusion and exclusion.

A total of 117 kidney transplant recipients were selected at the LUMC between 2010 and 2021. These patients received a kidney from a deceased donor and developed BKPyV DNAemia within the first year post-transplantation. In addition, 133 recipients of a deceased donor kidney transplanted between 2018 and 2021 who did not develop BKPyV DNAemia during follow-up were included. From this combined cohort of 250 recipients, 106 patients were excluded because the contralateral kidney was either not transplanted or was transplanted outside the Netherlands. Another 15 patients were excluded because the contralateral kidney was transplanted in a Dutch academic center that did not participate in the study. Of the remaining cases, 10 were excluded due to primary non-function of the contralateral kidney, 1 patient was excluded due to lack of follow-up, and 1 patient was excluded due to early post-transplant mortality before BKPyV screening could be performed. This resulted in a final cohort of 117 recipient pairs (Figure 1). The contralateral kidneys in these pairs were transplanted across five different Dutch transplant centers: 28 at the UMCG, 32 at the EMC, 7 at the UMCU, 9 at the MUMC+, 33 at the AUMC, and 8 at the LUMC. The 117 contralateral kidney transplant (KTx) recipients were followed for an average of 328 days (range: 59–366 days) until the occurrence of BKPyV DNAemia, graft loss, mortality, or the maximum follow-up period of 1 year.

In the LUMC group, baseline patient characteristics were largely comparable between recipients who developed BKPyV DNAemia and those who did not. In the BKPyV DNAemia group, 4 patients were on a steroid-free regimen as part of a clinical study that included alemtuzumab induction (Table 1).

In the contralateral group, patients who developed BKPyV DNAemia experienced more DGF, were more likely to have pre-existing HLA antibodies, with a PRA above 5%, and were more frequently treated with immunosuppressive regimens based on ciclosporin. In contrast, those who did not develop BKPyV DNAemia were more frequently initiated on everolimus-based immunosuppression (Table 1).

When comparing the characteristics of BKPyV DNAemia, there was no significant difference in the duration, maximum measured serum viral load, or incidence of BKPyVAN between the contralateral group with BKPyV DNAemia and the LUMC group with BKPyV DNAemia. However, the onset of BKPyV DNAemia occurred significantly later in the contralateral group compared to the LUMC group (Table 1).

The incidence of BKPyV DNAemia among recipients whose contralateral kidney recipient also developed BKPyV DNAemia was significantly higher compared to those whose contralateral recipient did not (28.9% [15/52] vs. 10.8% [7/65], *p* = 0.013). In univariate Cox regression analysis, BKPyV DNAemia in the contralateral recipient was associated with an increased risk of BKPyV DNAemia, with a hazard ratio (HR) of 3.0 (95% CI: 1.2–7.3; *p* = 0.018). In multivariate Cox regression analysis, the final model included the development of BKPyV DNAemia in contralateral recipients, PRA, induction therapy, maintenance immunosuppression, and recipient and donor age. After adjustment for these factors, BKPyV DNAemia in the contralateral recipient remained significantly associated with an increased risk of BKPyV DNAemia (HR 4.9, 95% CI: 1.8–13.6; *p* = 0.002) (Table 2).

### Sensitivity Analysis Results

Two sensitivity analyses were performed. First, patients transplanted at the MUMC+ were excluded because of a less intensive immunosuppressive strategy. This analysis included 108 recipient pairs. In both univariate (HR 3.2, 95% CI 1.3–7.9; *p* = 0.013) and multivariable Cox regression analysis (HR 4.8, 95% CI 1.5–15.7; *p* = 0.010), development of BKPyV DNAemia was significantly associated with BKPyV DNAemia in the contralateral recipient. Second, an additional analysis was performed, including only patients transplanted between 2018 and 2021. This analysis included 89 recipient pairs. The association remained significant in both univariate (HR 3.0, 95% CI 1.1–8.5; *p* = 0.041) and multivariable analysis (HR 4.8, 95% CI 1.5–15.7; *p* = 0.010).

## 4. Discussion

This study shows an increased risk for the development of BKPyV DNAemia in recipients of a deceased kidney donor when the recipient of the contralateral kidney develops BKPyV DNAemia. This underscores the influence of donor-derived factors in the transmission of BKPyV in kidney transplantations from deceased donors.

This study is among the largest to demonstrate the influence of donor-related factors in kidney transplant recipients. Previously, Lorentzen et al. reported identical viral DNA sequences in plasma samples from two kidney recipients of the same deceased donor, both of whom developed BKPyVAN [11]. Similarly, Bohl et al. demonstrated that 16 out of 20 recipient pairs of a postmortem donor were concordant [12]. Furthermore, in a large registry study including over 20,000 paired kidney transplants, Thangaraju et al. found that paired BKPyV infections occurred 2.8 times more frequently than expected based on the overall incidence [13]. In association with our findings, these studies emphasize the significant role of donor-derived factors in the risk of BKPyV infection.

The exact pathophysiological mechanism of donor-derived BKPyV infection remains unclear. Seroprevalence of BKPyV exceeds 90% in the healthy population, indicating that most kidney transplant recipients are likely to have some degree of pre-existing immunity against the virus [14,15]. Nevertheless, it is possible that the recipient may acquire a new BKPyV subtype from the donor, as some cases have documented a subtype switch in recipients following transplantation [12]. However, this does not account for all cases. For example, both Bohl et al. and Wunderink et al. observed that both donors and recipients predominantly harbored subtype I BKPyV [12,16]. However, by focusing solely on the large T-antigen (LTag) for BKPyV subtyping, other mutations in minor viral strains may be overlooked, even though these mutations can significantly influence viral virulence [16]. Additionally, Leuzinger et al. have shown that small amino acid variations within the LTag can alter how viral epitopes are presented by HLA class I molecules, thereby affecting CD8^+^ T-cell recognition and the immune response to BKPyV [17]. Another potential explanation involves viral load, since there is an association between higher seroreactivity in the donor and an increased risk of BKPyV-related infection in the recipient [9]. This finding suggests a high viral load in the donor as a contributing factor to infection risk in recipients. Further research is needed to clarify the underlying mechanism.

Given that BKPyV DNAemia can cause graft damage not only through BKPyVAN but also due to its association with rejection [18,19,20,21,22] and that donor factors appear to influence its development, it is essential to explore therapeutic strategies beyond simply reducing immunosuppression, which remains the current standard of care [23,24]. Potential strategies include screening donors for BKPyV serotypes and seroresponse, as Solis et al. have shown that mismatches between genotype-specific neutralizing antibodies and the corresponding BKPyV strain are associated with an increased risk of BKPyV-related infection, whereas the presence of high neutralizing antibody titers against the specific BKPyV genotype confers protection. These strategies could then facilitate better donor-recipient matching, identify recipients who may benefit from intensified post-transplant monitoring, or guide tailored immunosuppressive regimens to reduce the risk of BKPyV-related complications [25]. Additionally, vaccinations against BKPyV could provide a preventive measure to enhance immune control of the virus and reduce the risk of subsequent BKPyV-related complications. Alongside vaccination, broadly neutralizing and monoclonal antibodies targeting BKPyV are currently being explored as both therapeutic and pre-emptive strategies [26]. Whether these approaches are effective will need to be determined in ongoing and future studies.

This study’s strengths include its large sample size and multicenter design, enhancing the generalizability of our findings. However, several limitations must be acknowledged. Firstly, the substantial variability in BKPyV screening protocols across centers raises the possibility that episodes of BKPyV DNAemia could have been missed in both recipients. Furthermore, we restricted our analysis to BKPyV DNAemia within the first year post-transplantation. While the first year represents the peak incidence of BKPyV, we did not examine the potential impact of BKPyV DNAemia occurring beyond this period. Additionally, variations in PCR methods and detection limits between centers may have influenced BKPyV DNAemia detection, potentially leading to missed cases in some centers. Urinary BKPyV testing was not systematically performed in the participating centers, precluding assessment of viruria. In addition, no suitable samples were available for viral sequencing, preventing further molecular confirmation of whether the viruses in recipient pairs were identical.

The retrospective design limits full adjustment for all potential confounders, particularly inter-center differences in immunosuppressive regimens and calendar-time effects. However, sensitivity analyses excluding MUMC+ recipients and restricting the cohort to transplants performed between 2018 and 2021 yielded consistent results, with the association remaining statistically significant. In addition, in a previously described cohort from our center, the incidence of BKPyV DNAemia within the first year after transplantation remained stable over time (2011–2013: 19.9%; 2014–2017: 20.0%; 2018–2020: 22.9%; *p* = 0.595), supporting the assumption that calendar-time effects did not meaningfully influence the observed associations [20].

## 5. Conclusions

In conclusion, our findings emphasize the significant role of donor factors in the development of BKPyV DNAemia in recipients of kidney transplants. Future research should focus on improving donor screening to enable personalized strategies for the prevention of donor-derived BKPyV.

## Figures and Tables

**Table 1:** Patient characteristics of kidney transplant recipients from deceased donors at the LUMC and contralateral recipients, stratified by BKPyV DNAemia status.

	Transplanted at LUMC Leiden			Contralateral Recipients		
	No BKPyV DNAemia (n: 65)	BKPyV DNAemia (n: 52)	*p*-Value	No BKPyV DNAemia (n: 95)	BKPyV DNAemia (n: 22)	*p*-Value
**Age,** years (mean ± SD)	62.3 (10.1)	61.2 (11.6)	0.579 **	57.5 (11.8)	57.9 (11.6)	0.972 **
**Gender,** male (n, %)	45 (69.2%)	38 (73.1%)	0.649 *	50 (52.6%)	14 (63.6%)	0.350 *
**Donor age,** years (mean ± SD)	57.8 (11.7)	57.4 (11.9)	0.853 **	57.5 (11.8)	57.9 (11.6)	0.861 **
**PRA pretransplantation** (immunized >5%) (n, %)	11 (16.9%)	9 (17.3%)	0.999 †	4 (4.3%)	6 (27.3%)	**0.003** †
**Induction** (n, %)			0.999 †			0.999 †
**Basiliximab**	63 (96.9%)	50 (96.2%)		86 (90.5%)	20 (90.9%)	
**Alemtuzumab**	2 (3.1%)	2 (3.8%)		2 (2.1%)	1 (4.5%)	
**No induction**				7 (7.4%)	1 (4.5%)	
**Maintenance Immunosuppression** (n, %)			0.005 †			**0.027** †
**Tac/CC/Pred**	51 (79.7%)	39 (75.0%)		91 (95.8%)	20 (90.9%)	
**Cicl/CC/Pred**	6 (9.4%)	9 (17.3%)		0 (0%)	2 (9.1%)	
**Tac/EVL/Pred**	2 (3.1%)	0 (0%)		4 (4.2%)	0 (0%)	
**CC/Tac**	0 (0%)	4 (7.7%)		0 (0%)	0 (0%)	
**Bela/CC/Pred**	5 (7.8%)	0 (0%)		0 (0%)	0 (0%)	
**Delayed Graft function** (n, %)	28 (43.1%)	21 (40.4%)	0.769 *	34 (35.8%)	13 (59.1%)	**0.045** *
**BKPyV DNAemia Characteristics**						
**Onset after transplantation,** days (mean ± SD)		119 (72)			177 (136)	**0.018** **
**Duration BKPyV DNAemia,** days (mean ± SD) ^^^		370 (510)			234 (380)	0.267 **
**Maximum BKPyV DNAemia, log10 copies/mL,** (mean ± SD)		4.1 (1.3)			3.9 (1.2)	0.469 **
**BK nephropathy** (n, %)		2 (3.8%)			2 (9.1%)	0.577 †

*p*-Values were calculated using the * chi-square test, ** independent-sample *t*-test, or † Fisher’s exact test. *p*-Values < 0.05 were considered statistically significant and are presented in bold. The table shows only variables with significant differences or those included in the final multivariable Cox regression model. Candidate variables not retained in the final model or not statistically significant are not shown (e.g., donor type, ureteral stent usage and donor gender).Abbreviations: BKPyV, BK polyomavirus; LUMC, Leiden University Medical Center; PRA, panel reactive antibody; SD, standard deviation; Tac, tacrolimus; CC, mycophenolate mofetil; Pred, prednisone; Cicl, cyclosporine; EVL, everolimus; Bela, belatacept. ^^^ Duration of BKPyV DNAemia was counted until two consecutive negative BKPyV serum loads.

**Table 2:** Association between BKPyV DNAemia in the LUMC recipient and the risk of BKPyV DNAemia in contralateral kidney transplant recipients.

	BKPyV DNAemia LUMC (n = 52)	No BKPyV DNAemia LUMC (n = 65)	*p*-Value
BKPyV DNAemia in contralateral recipient (n, %)	15 (28.8%)	7 (10.8%)	**0.013** *
**Cox regression analysis**			
Univariable	3.0 (1.2–7.3)	reference	**0.018** **
Multivariable	4.9 (1.8–13.6)	reference	**0.002** ***

* Chi-square test was used to compare the incidence of BKPyV DNAemia dependent on the BKPyV DNAemia status of contralateral recipients. Hazard ratios (HRs) were calculated with uni- and multivariable Cox regression analysis; ** describes the HR univariate analysis of BKPyV DNAemia in recipients where the contralateral recipient developed BKPyV DNAemia within the LUMC, compared to cases in which the contralateral recipient did not develop BKPyV DNAemia; *** describes the multivariable analysis HR, corrected for the following factors: PRA, induction therapy, maintenance immunosuppression, and recipient and donor age. *p*-values < 0.05 and are presented in bold were considered statistically significant. Abbreviations: BKPyV, BK polyomavirus; LUMC, Leiden University Medical Center, HR, hazard ratio; n, number.

## Data Availability

The individual-level clinical data underlying this study cannot be shared publicly due to ethical and privacy restrictions, as approved in the local non-WMO declaration by the institutional review board (Head of the Department of Internal Medicine—Scientific Committee) of the Leiden University Medical Center (LUMC). Specifically, data sharing with external parties was not permitted under the terms of approval for this retrospective study. The data contain potentially identifying patient information, and full anonymization is not feasible due to the detailed nature of clinical and transplant-related variables. Researchers may request access to de-identified, aggregated data or specific analyses performed by the study team, subject to approval by the LUMC Data Access Committee and in compliance with the General Data Protection Regulation (GDPR) and institutional policies. Requests should be directed to the corresponding author.

## Abbreviations

BKPyV	BK polyomavirus
BKPyVAN	BK polyomavirus-associated nephropathy
LUMC	Leiden University Medical Center
ET	Eurotransplant
NTS	Nederlandse Transplantatie Stichting
UMCG	University Medical Center Groningen
UMCU	University Medical Center Utrecht
MUMC+	Maastricht University Medical Center
EMC	Erasmus Medical Center Rotterdam
AUMC	Amsterdam University Medical Center
SV40	Simian virus 40
dnDSA	Donor-specific antibodies
DBD	Donation after brain death
DCD	Donation after circulatory death
DGF	Delayed graft function
HLA	Human leukocyte antigen
qPCR	Quantitative real-time polymerase chain reaction
EDTA	Ethylenediaminetetraacetic acid
PRA	Panel-reactive antibody
HR	Hazard ratio
CI	Confidence interval
KTx	Kidney transplantation
LTag	Large T-antigen
